# The complete mitochondrial genome of *Microconidiobolus nodosus* (*Entomophthorales*: *Ancylistaceae*)

**DOI:** 10.1080/23802359.2021.1930219

**Published:** 2021-05-23

**Authors:** Yue Cai, Yong Nie, Zi-Min Wang, Bo Huang

**Affiliations:** a College of Biology, Food and Environment, Hefei University, Hefei, China; b Anhui Provincial Key Laboratory for Microbial Pest Control, Anhui Agricultural University, Hefei, China; c School of Civil Engineering and Architecture, Anhui University of Technology, China

**Keywords:** *Microconidiobolus*, entomophthoroid fungus, mitochondrion, phylogeny

## Abstract

In this study, the complete mitochondrial genome of *Microconidiobolus nodosus* was sequenced which is the first mitochondrial genome of the genus. The mitochondrial genome is 31,638 bp long and 27.18% in GC ratio, and it contains 14 conserved protein-coding genes, 2 ribosomal RNAs and 22 transfer RNAs. Phylogenetic analysis showed that *M. nodosus* was closely related to *Conodiobolus* sp. This study reported the whole mitochondrial genome and character of a basal fungus *M .nodosus* and provided a better understanding of the phylogeny of basal fungi.

The genus *Neoconidiobolus* B. Huang & Y. Nie (2020) was established to accommodate three species producing smaller primary conidia (mostly less than 20 μm) than other *Conidiobolus* spp. and producing neither microspores nor capilliconidia (Nie et al. 2020). In the previous study, two complete mitochondrial genomes of *Capillidium heterosporum* (Drechsler) B. Huang & Y. Nie (2020) and *Conidiobolus* sp. have been reported, and their phylogenetic status was also confirmed in the lineage of *Entomophthoromycotina*.

The ex-type *Microconidiobolus nodosus* (Sriniv. & Thirum.) B. Huang & Y. Nie (2020) strain ATCC 16577 was obtained from American Type Culture Collection (Manassas, USA). The genomic DNA was extracted from the mycelia using the CTAB method (Watanabe et al. 2010). The whole genome of *M. nodosus* was sequenced on an Illumina HiSeq X Ten Platform (Pacific Biosciences, Nextomics Biosciences, Co., Ltd., Wuhan, China). Low-quality bases at the ends of the sequence reads were trimmed off by the quality control and the mitogenome was assembled by NOVOPlasty (Dierckxsens et al. 2017) by enabling the option to circularize contigs with matching ends. The mitogenome annotation was performed with MFannot tool (http://megasun.bch.umontreal.ca/cgi-bin/mfannot/mfannotInterface.pl) using the mitochondrial genetic code (genetic code 4) (Zhang et al. 2017). Transfer-RNA annotations were further predicted and confirmed by tRNAscan-SE 1.3.1 (Lowe and Eddy 1997).

The mitogenome of *M. nodosus* (GenBank accession number MW_795365) is 31,638 bp long with the GC content of 27.18%. It contains a set of 28 protein-coding genes, 2 rRNA genes (rns and rnl) and 23 tRNA genes. Among the 28 protein-coding genes, 14 protein-coding genes were core protein-coding genes: *cob*, *cox1*, *cox2*, *cox3*, *nad1*, *nad2*, *nad3*, *nad4*, *nad4L*, *nad5*, *nad6* and *atp6*, 13 free-standing ORFs and a gene homologous to *rps3*. Seven introns were found in protein-encoding genes, including 1 in *nad5*, 2 in *cob* and 4 in *cox1*.

The phylogeny was inferred by the concatenated amino acid sequences of 14 proteins encoded by the conserved mitochondrial genes. The accession numbers of species used for evolutionary analysis were: *Allomyces macrogynus* (NC_001715), *Blastocladiella emersonii* (NC_011360), *Capillidium heterosporum* (NC_040967), *Conidiobolus* sp. (MN_640580), *Hirsutella minnesotensis* (NC_027660), *Lactifluus hygrophoroides* (NC_038206), *Lichtheimia hongkongensis* (NC_024200), *Monoblepharella* sp. (NC_004624), *Pestalotiopsis fici* (NC_031828), *Podila verticillata* (NC_006838), *Rhizophagus fasciculatus* (NC_029185), *Spizellomyces punctatus* (NC_003052), *Tremella fuciformis* (NC_036422), *Zancudomyces culisetae* (NC_006837). *Drosophila melanogaste*r (NC_024511) and *Mielichhoferia elongata* (NC_036945). The sequences of 14 proteins were locally aligned with BioEdit (Hall 1999) and concatenated with SequenceMatrix (Vaidya et al. 2011). The final alignment length was 5,494 nucleotides and the model GTR + G + I was determined to be the most suitable evolutionary model (Li et al. 2021). The phylogeny was reconstructed using maximum likelihood in RAxML 8.1.17 (Stamatakis 2014). *Microconidiobolus nodosus* was grouped with *Capillidium heterosporum* and *Conidiobolus* sp. as the representative of *Entomophthoromycotina* in a well-supported clade (Figure 1). This result was congruent with the previous studies (Nie et al. 2019; Sun et al. 2020).

**Figure 1.**
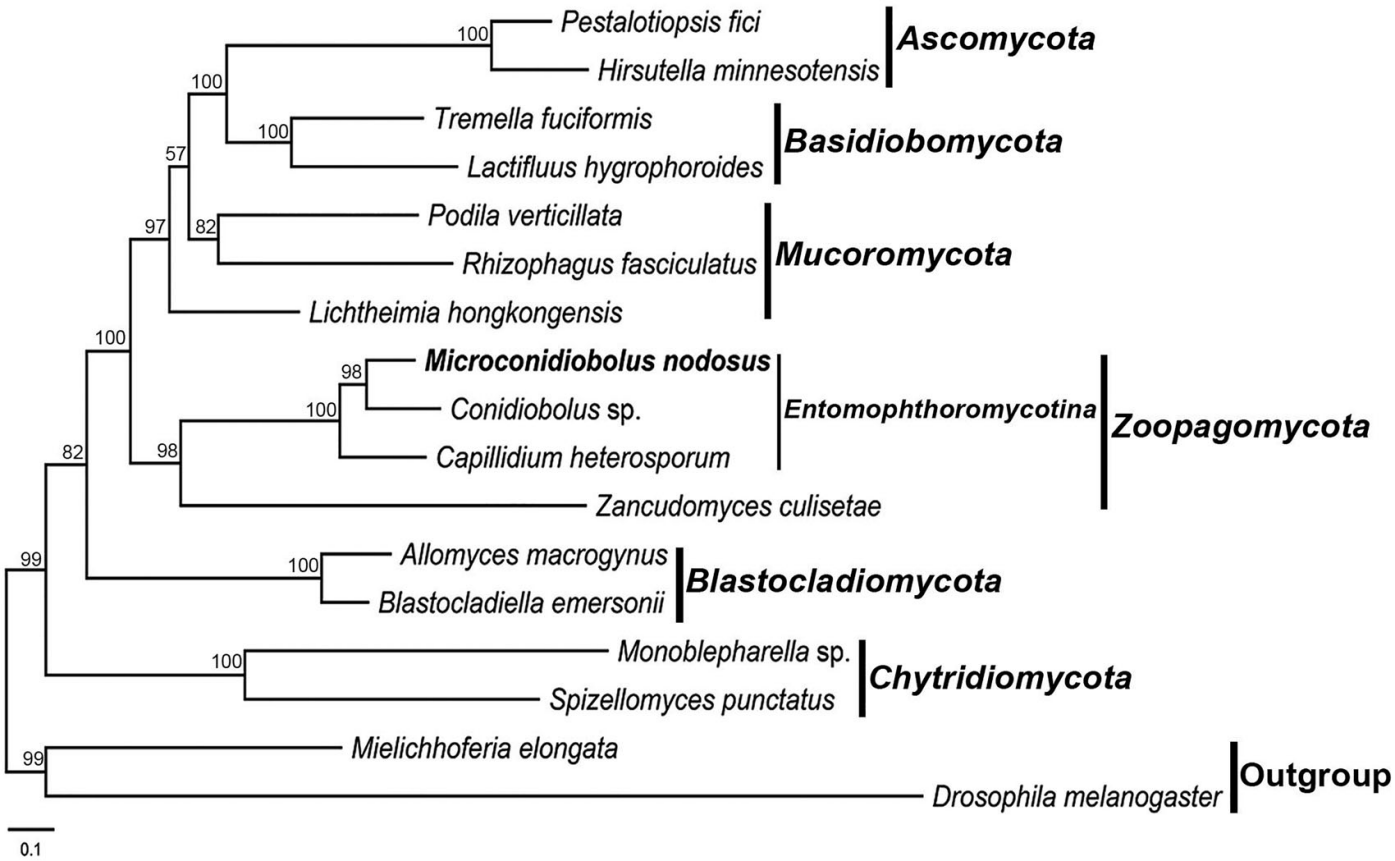
Phylogenetic tree constructed by maximum likelihood analyses based on 14 translated mitochondrial proteins. They included oxidase subunits (*Cox1*, *2*, and *3*), the apocytochrome b (*Cob*), ATP synthase subunits (*Atp6*, *Atp8*, and *Atp9*), NADH dehydrogenase subunits (*Nad1*, *2*, *3*, *4*, *5*, *6*, and *Nad4L*). The 14 fungal mitogenomes were used in this phylogenetic analysis. *Drosophila melanogaster* and *Mielichhoferia elongata* were served as outgroups. Maximum likelihood bootstrap values (500 replicates) of each clade are indicated along branches. Scale bar indicates substitutions per site.

## Data Availability

The data that support the findings of this study are openly available in NCBI (https://www.ncbi.nlm.nih.gov/). The accession number is MW_795365.
